# *Journal of Physiological Anthropology *aims to investigate, how the speed of technological advance, experienced during the 21^st ^century, is affecting mankind

**DOI:** 10.1186/1880-6805-31-1

**Published:** 2012-02-29

**Authors:** Akira Yasukouchi

**Affiliations:** 1 Department of Physiological Anthropology, Faculty of Design, Kyushu University, Fukuoka, Japan

## Editorial

*Journal of Physiological Anthropology *(*JPA*) covers a broad range of studies on humans living in modern society. Today's highly technological environment has developed rapidly, when we consider the time scale of human history, and rapid advances in science and technology are thought to be exerting profound effects on the human community, in terms of not only lifestyle and culture, but also the physiological capabilities of the human body. Against this background, *JPA *presents research on humans in modern society, evaluating humankind mainly from a physiological perspective, in efforts to create truly healthy and adaptable living environments. Our general interest is focused on what it is that mankind seeks. What sorts of selective pressures are exerted upon modern humans, and what sorts of responses do these pressures elicit? What biological strategies do humans use to adapt flexibly to ever changing modern environments? In the discipline of physiological anthropology, which focuses on human adaptability to the environment and its diversity, our research is centered around the key concepts of physiological polytypism; the potential capabilities of physiological traits (functional potentiality); physiological coordination of the whole body (whole-body coordination); environmental adaptability and techno-adaptability.

The maximum functional ability of mankind has both manifest and latent components and the boundary between these two components changes depending on the type, frequency and intensity of stress encountered in daily life, which are in turn dependent upon the behavioral history of individuals. The three basic concepts of physiological polytypism, functional potentiality and whole-body coordination can be briefly defined as follows. Physiological polytypism is a characteristic phenotype of the manifest component and it depicts a specific difference manifested by an individual. Although physiological polytypism is influenced by gene and environmental factors, the latter effects, especially, are of a more plasticizing nature. Functional potentiality defines the superficially non-apparent, or latent, component of maximum functional ability. In these terms a coordinated response is considered to involve the body's functional adjustment system, which maintains homeostasis against certain stresses. The adjustment system is constructed and affected by the degree of the manifested function and the output distribution of various sub-system or element functions. These together mold the specific patterns of coordinated responses, and where the coordination system is systemic, it is known as whole-body coordination.

We can use the three concepts of physiological polytypism, functional potentiality and whole-body coordination to discuss environmental adaptability and techno-adaptability from the viewpoint of the adaptability of groups, thereby allowing us to consider the nature of human diversity and its meaning.

The elements affecting phenotype in polytypism include gene, physical environmental and culture related factors. The phenotype is affected by genes themselves as well as by the interactions of physical and/or cultural factors on the genes [1]. Since our lifestyles and the environments in which we live are consistently shifting and changing with the advances made in science and technology, we need to adapt to these changes in our living environment. Therefore, the factors influencing phenotype are more likely to be physical and cultural, and their interactions with genes are more likely to involve gene-environment interplay, when considering several generations only. In this sense, the term *adaptation *refers to phenotypic plasticity. This is one of reasons why *JPA *focuses on physiological plasticity.

*JPA *was first published in 1983 as the official journal of the Japan Society of Physiological Anthropology, under the name of *Annals of Physiological Anthropology*. It was later renamed the *Applied Human Science Journal of Physiological Anthropology *(1995-1999) and then the *Journal of Physiological Anthropology and Applied Human Science *(2000-2005) when it became an English language journal. *JPA *has existed in its present form since 2006. In January 2010, *JPA *was selected for impact factor tracking by Thomson Reuters, the first impact factor is due in 2013.

With the release of its 31^st ^volume, *JPA *is being re-launched in 2012 as an open access journal. *JPA *will now be published online by BioMed Central. Whilst *JPA*'s abstracts are currently archived in PubMed, following the move to BioMed Central, articles will be included in many freely accessible full-text repositories. Additionally the peer review process will be completed using BioMed Central's online submission system and will mainly be undertaken by our 16 Editors and 46 member Editorial Board consisting of internationally renowned researchers. More detailed information about the journal and the Japan Society of Physiological Anthropology can be found in references [2-5].

Open access (OA) to academic journals has increased in recent years, with 8.5% [6] of peer-reviewed publications currently offering full OA. Although *JPA *is already available in OA format via the Japan Science and Technology Agency (JST) online database, the decision to publish with BioMed Central seeks to harness its immense and uniquely branded publicity as the world's leading OA publisher. This will enable *JPA *to reach a wider international audience [7] and opportunities to strive for increasing impact factors post 2013.

BioMed Central has the ability to effectively enhance *JPA*'s global visibility through its highly developed websites and extensive network of over 1 million registered users, and reaches out via the use of electronic media including email, Facebook, Twitter and newsletters. Moreover, BioMed Central's online submission system enables effective peer review and allows authors to better understand reviews of their work, while its policy of distributing articles soon after acceptance is expected to dramatically reduce the time from manuscript receipt to publication. The online publication format will also eliminate the cost of color printing and document length limitations.

The Editorial Board will endeavor to enhance *JPA*'s reputation as a quality, relevant publication striving to gain and subsequently increase the impact factor. We look forward to receiving many articles from you in the future.
